# Cascading tipping points of Antarctica and the Southern Ocean

**DOI:** 10.1007/s13280-024-02101-9

**Published:** 2024-12-10

**Authors:** Ida Kubiszewski, Vanessa M. Adams, Rachel Baird, Anne Boothroyd, Robert Costanza, Darla Hatton MacDonald, Glenn Finau, Elizabeth A. Fulton, Catherine K. King, Matt A. King, Delphine Lannuzel, Elizabeth Leane, Jess Melbourne-Thomas, Can-Seng Ooi, Mala Raghavan, Valeria Senigaglia, Natalie Stoeckl, Jing Tian, Satoshi Yamazaki

**Affiliations:** 1 Institute for Global Prosperity, University College London, London, UK; 2 College of Business and Economics, University of Tasmania, Hobart, Australia; 3 School of Geography, Planning, and Spatial Sciences, University of Tasmania, Hobart, Australia; 4 College of Arts, Law and Education, University of Tasmania, Hobart, Australia; 5 CSIRO Environment, Hobart, TAS Australia; 6 Centre for Marine Socioecology, Hobart, TAS Australia; 7 Environmental Stewardship Program, Australian Antarctic Division, Kingston, TAS Australia; 8 Australian Centre for Excellence in Antarctic Science, University of Tasmania, Hobart, Australia; 9 Institute for Marine and Antarctic Studies, University of Tasmania, Hobart, Australia; 10 School of Social Sciences, University of Tasmania, Hobart, Australia; 11 Securing Antarctica’s Environmental Future, School of Mathematical Sciences, Queensland University of Technology, Brisbane, Australia

**Keywords:** Antarctic treaty system, Earth system, Ecosystem services, Interacting tipping points, Ocean circulation, Sea ice

## Abstract

Antarctica and the Southern Ocean are key elements in the physical and biological Earth system. Human-induced climate change, and other human activities in the region, are leading to several potential interacting tipping points with major and irreversible consequences. Here, we examine eight potential physical, biological, chemical, and social Antarctic tipping points. These include ice sheets, ocean acidification, ocean circulation, species redistribution, invasive species, permafrost melting, local pollution, and the Antarctic Treaty System. We discuss the nature of each potential tipping point, its control variables, thresholds, timescales, and impacts, and focus on the potential for cumulative and cascading effects as a result of their interactions. The analysis provides substantial evidence of the need for more concerted and rapid action to limit climate change and to minimise the impacts of local human activities to avoid these cascading tipping points.

## Introduction

The Antarctica and Southern Ocean (A&SO) is one of the most remote parts of our planet. It is also one of the most important for climatic and oceanic regulation and stability (Bennett et al. 2015). We use the Antarctic Convergence, located approximately 55° South, to define the northern boundary of this region for ecosystem processes associated with Antarctic water masses. For social processes, we use the Antarctic Treaty System (ATS) which defines the A&SO to latitude 60° South.

Antarctica has a critical role in the circulation and exchange of heat, salt/freshwater, oxygen and nutrients throughout the world’s oceans (Goosse and Fichefet 1999). It also impacts atmospheric temperature gradients, heat transport, air chemistry, and rainfall patterns globally (Kennicutt et al. 2014). These global patterns help maintain global food webs and overall marine and terrestrial ecosystems.

Nevertheless, A&SO is experiencing major changes due to both local and global anthropogenic stressors (Chown and Brooks 2019). Changing climate, resource exploitation, and increases in local human activities are currently the most significant drivers of change (Convey and Peck 2019). Until now, these changes in Antarctica have had limited impact on the Earth system. However, with an increase in extreme events in Antarctica, and around the world, this may not last (Siegert et al. 2023). Ongoing pressures and small perturbations may lead to a drastic shift in the function of the Antarctic and global systems.

Dramatic local, regional, and global shifts, or tipping points, have become a growing research topic and a concern for policymakers (Russill and Nyssa 2009; Russill and Lavin 2012; Lenton et al. 2022). Tipping points, driven by either local or global human activity, have been defined as critical thresholds within tipping elements triggered by small changes that can lead to large, often irreversible changes in the state of a system (Lenton et al. 2008; Milkoreit et al. 2018). In this paper, we define a tipping point similarly to Lenton et al. (2008) as a relatively sudden change in a tipping element, bringing the system into a new stable state that is not easily reversible. This new state is reached by transitioning through points of comparative instability. Tipping points occur in physical, biological, chemical, and social systems. The system may be brought close to the critical threshold by multiple factors. One example is a biophysical system like the Great Barrier Reef. Both climate change and local agricultural run-off (dependent on local environmental regulations) contribute to tipping the system from a coral to an algae-dominated state (Bohensky et al. 2011).

**Fig. 1:**
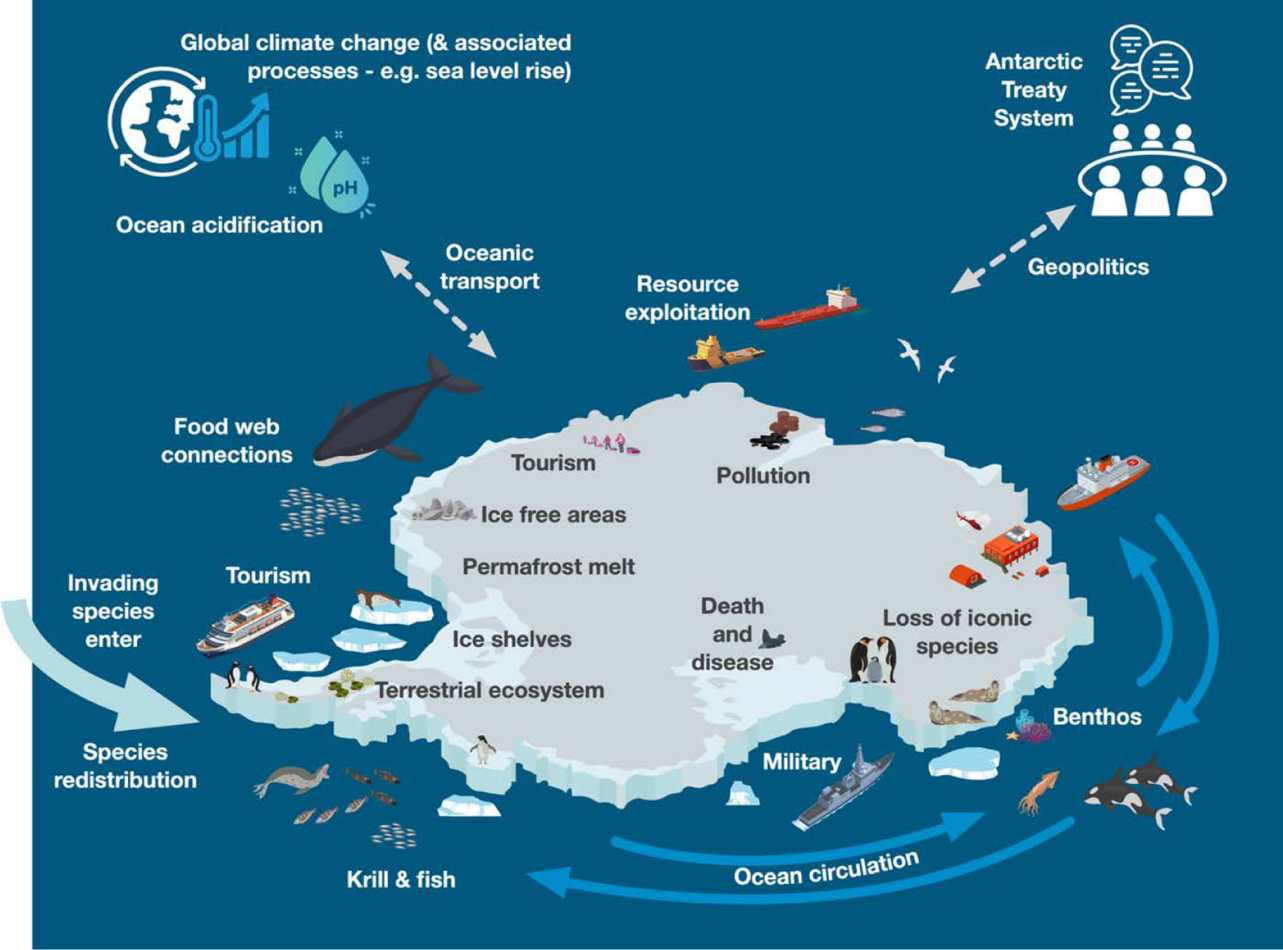
This figure shows the relationship of the two physical (ice sheets, and ocean circulation), three biological (species redistribution, invasive species, and permafrost), two chemical (ocean acidification and pollution), and one social (Antarctic Treaty System) tipping points in Antarctica and the Southern Ocean (A&SO). Figure credit: This figure was created by Derek Fulton and shutterstock artist A7880S

**Fig. 2:**
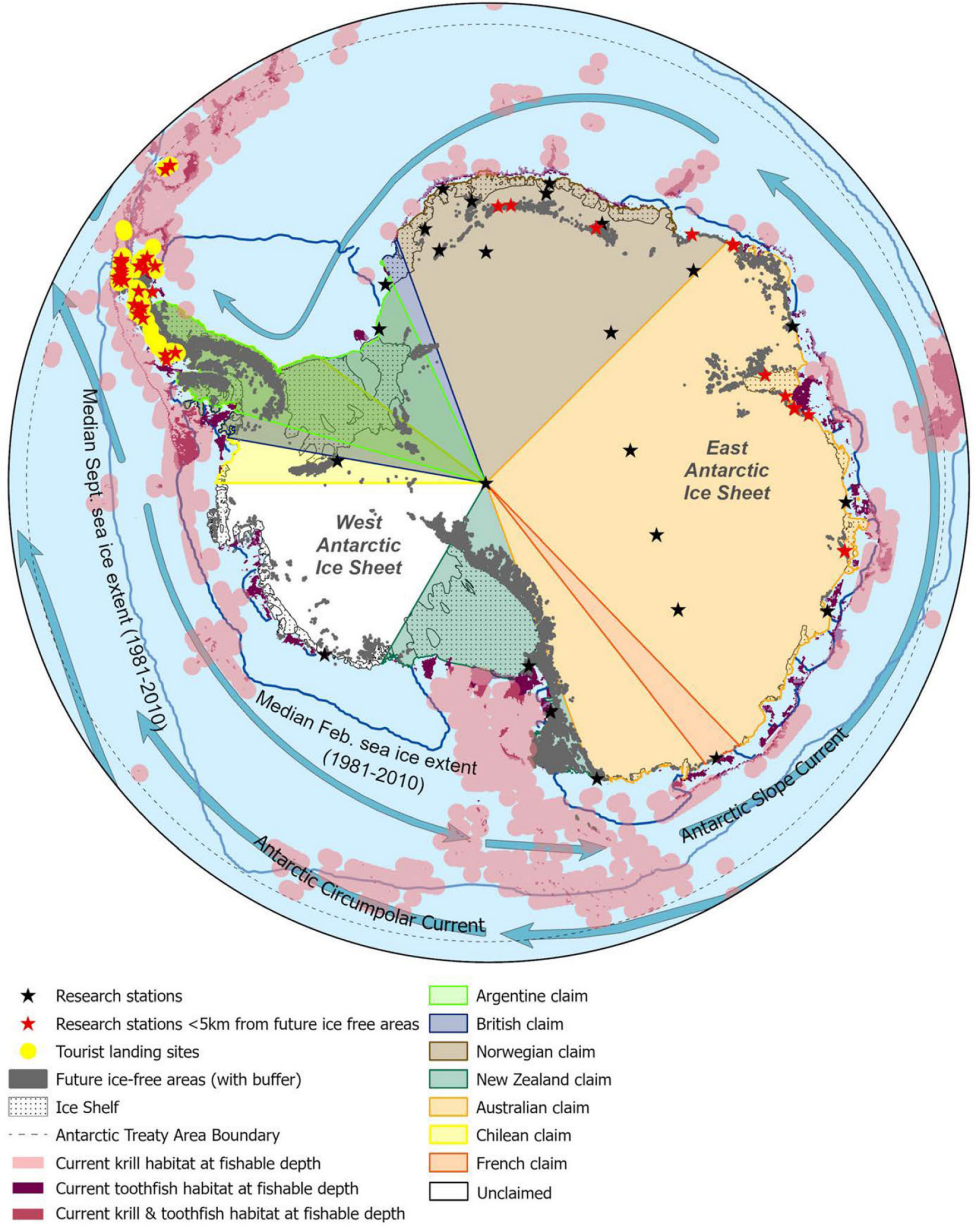
Map of Antarctica and the Southern Ocean. This map shows the location of human activities and the various potential Antarctic tipping point elements. It extends to 60° South, as recognised by the Antarctic Treaty System. Ice-free areas, tourist landing sites, and coastal research stations within 5 km of future ice-free areas, are most susceptible to pollution and the introduction of invasive species. The ice shelves show where the ice sheets are most likely to retreat and the ice shelves to collapse. The Treaty boundary indicates the management area for the ATS and its scope. The current krill and toothfish habitats at fishable depths indicate habitats that can be accessed by fisheries. These may be impacted by species redistribution, ocean acidification, sea ice melt, and pollution. The highlighted claims, research stations, and tourist sites show the location of significant human activity in Antarctica, which is governed by the ATS. The northern boundaries of the claims are not specified as Norway and Chile have no specified northern limit. The sea ice winter and summer extents, labelled on the map, show the large and variable area that the sea ice covers. The labelled currents show a critical part of oceanic circulation. *Data sources*: Research stations 5 km from future ice-free areas and tourist landing sites: Duffy and Lee 2019; Future ice-free areas; Lee and Terauds 2017; Coastline, Ice Shelf, Antarctic Treaty Area Boundary 60 degrees south: Esri 2014; Exploitable krill and toothfish habitat based on bathymetry: GEBCO 2021, Cuzin-Roudy et al. 2014; Sea ice: Guillaumot et al. 2018; Currents: ArcGIS.com 2016; Sea ice extent: Fetterer et al. 2017

In this paper, we discuss two physical (ice sheets, and ocean circulation), three biological (species redistribution, invasive species, and permafrost), two chemical (ocean acidification and pollution), and one social (Antarctic Treaty System) tipping points in Antarctica (Fig. 1). These eight tipping points independently can have dire consequences for A&SO, if not the globe. However, their complex interactions, whether synergistic, additive, or antagonistic, might cause more important cascades and should be taken into consideration. Figure 2 shows the spatial dimension of human activity considered as tipping elements, and associated physical characteristics which are critical to these tipping points in the A&SO. We also consider the timescale of these tipping points, at what thresholds the elements might tip, and the impacts they would have on both Antarctica and the globe (Table 1). Additionally, we look at how the tipping points could cascade. If one of the elements tips, how could that affect the tipping of the other elements? We also delve into the social and economic implications of these tipping points, discussing how they could impact each other and existing international agreements governing human interaction with Antarctica. By doing so, we aim to provide an enhanced understanding of the potential future of Antarctica in the face of ongoing climate change and increasing human activity. This paper aims to show that the governance of the region can adapt to limit further ecological degradation and potential tipping point cascades.

**Table 1:** Key elements of the Antarctic tipping points

Tipping elements	Control variables	Variable that is changing (direction of)	Threshold	When tipping	Transition timeframe (rapid/gradual/slow)	Impacts	Reversibility if Tipped	Sources
Ice sheet	Ocean and air temperature	Mass (-)	**West Antarctic Ice Sheet:** ~ 1.5 °C [1 to 3 °C] ••••◦ **East Antarctic Ice Sheet:** 3 °C [2 to 6 °C] •••◦◦	**West Antarctic Ice Sheet:** 2 ky [500 years to 13 ky]. Onset could have already commenced or in coming decades •••◦◦ **East Antarctic Ice Sheet:** 2 ky (500 y to 10 ky) •••◦◦	Gradual	**Global:** Sea level rise; ocean circulation (stratification) **Local:** Changes in local primary production; iceberg scouring; blocking of ships by icebergs and altered ocean depth; isolated or exposed infrastructure due to locally lowered or raised sea levels; expansion of ice-free areas; loss of endemic biodiversity & establishment of non-native species	Irreversible for millennia ••••◦	Armstrong McKay et al. (2022)
Ocean acidification	CO_2_ concentrations	Acidification ( +)	Approximately 450–600 ppm CO2 ••◦◦◦	30–100 years ••••◦	Gradual	**Global:** Reduction in CO2 sequestration **Local:** Loss of calcifying organisms; changes in food web; decline in fisheries	Irreversible for millennia ••••◦	Heinze et al. (2021), McNeil and Matear (2008)
Ocean circulation	Ice sheet melt, sea-ice formation, wind and heat flux	Density (-) Nutrient content (-)	− 42% (10.0 Sv) due to bottom water − 0.06 psu shift (due to melt water) •••◦◦ − 0.3 to − 16 µM drop in surface ocean nutrients outside the SO •••◦◦	30 years •••◦◦ Progressively over 250 years •••◦◦	Gradual or rapid Gradual	**Global:** Reduced heat, gas, oxygen transport **Local:** Decline in Antarctic bottom water formation, poleward shift of westerlies, increased ocean stratification **Global:** Reduced nutrient transport **Local:** Accelerated phytoplankton growth rates and longer growing seasons	Irreversible for millennia •••◦◦	Heinze et al. (2021), Li et al. (2023) Moore et al. (2018b)
Species redistribution	Ocean and air temperature	**Land:** Ice-free area ( +) & connectivity **Ocean:** Depth and area ( ±)	**Land:** Unknown **Ocean:** Between + 4 °C and + 10 °C ••••◦	**Land:** 2050–2100 ••◦◦◦ **Ocean:** 2100 •◦◦◦◦	Gradual	**Global:** Changes in global marine ecosystems; challenges in managing transboundary fish stocks; new pressures on the ATS **Local:** Displacement & loss of species; emergence of novel ecosystems; homogenization of ecosystem; emergence of novel ecosystems	Reversible in decades to centuries •••◦◦	Lee et al. (2017), Peck et al. (2014)
Invasive species	Human activity and long-distance biologically mediated travel	Number ( +)	1 key species, or many non-key species •◦◦◦◦	Unknown	Gradual or rapid	**Local:** Loss of endemic species; new ecosystem states	Reversible in years to decades ••◦◦◦	Chown et al. (2012a)
Permafrost	Air temperature	Soil temperature ( +)	+ 2 °C ••••◦	Before 2100 ••◦◦◦	Gradual Rapid if nonlinear response occurs	**Global:** Release of CO2, CH4, and He; release of pathogens that spread across polar food webs or enter human populations; **Local:** Release of viruses, bacteria, fungi, and other microorganisms;	Irreversible for centuries ••◦◦◦	Biskaborn et al. (2019)
Pollution	Human activity/long-range transport of contaminants/air temperature	Heavy metals, hydrocarbon fuels, organic pollutants, plastics ( +)	Dependent on contaminant volume and concentrations, toxicity to biota, and synergistic effects	Unknown. Could occur anytime if catastrophic fuel spill	Gradual Rapid in extreme contamination event	**Global:** Impacts on migratory seabird population **Local:** Bioaccumulation and biomagnification of pollutants; reduced local biodiversity and community change; introduction of non-native microbial species, viruses, bacteria, and parasites	Irreversible in decades ••◦◦◦	Stark et al. (2016), Tin et al. (2009)
Antarctic Treaty System	Development pressure / undermining confidence/ international conflict/ regional disputes	Diplomacy (-)	Unknown. The Environmental Protocol can be modified by consensus at any time. In 2048, a review can be called, but changes would need to be by majority ••◦◦◦	Unknown. The Environmental Protocol can be modified by consensus at any time. In 2048, a review can be called, but changes would need to be by majority ••◦◦◦	Slow Could be rapid if extreme circumstances arise	**Global:** Escalation of political tensions between countries; Local: Reduced management of living and non-living resources; reduced regulation of human activity;	Reversible in years to decades ••••◦	Rothwell (2021)

The data in this table is derived from sources stated in the text, the expertise of the co-authors, or indicated in the ‘Source’ column. The threshold temperatures provided are given as above pre-industrial levels. IPCC-style confidence levels are provided for thresholds and time of tipping (Mastrandrea et al. 2010). [Confidences: ••••• very high; ••••◦ high; •••◦◦ medium; ••◦◦◦ low; •◦◦◦◦ very low]

## The tipping points

### Ice sheet

The configuration of an ice sheet, or continental glacier, resting on bedrock below sea level and with deepening interior ice is inherently unstable and vulnerable to a tipping point behaviour known as the Marine Ice Sheet Instability (MISI) (Schoof 2007). Once a part of an ice sheet in this configuration begins to retreat from the grounding line, forced by ocean-driven melt increase, that part will experience a self-sustaining retreat even after the removal of the force that initially triggered the retreat. Such retreat may be accelerated by the collapse of buttressing ice shelves, possibly triggered by surface melt and hydrofracture, leading to unstable ice-cliff geometry and subsequent collapse. This is known as the Marine Ice Cliff Instability and is yet to be conclusively observed (e.g., DeConto and Pollard 2016; Bassis et al. 2024)). This retreat may last years, decades, or centuries and will progress until a new bedrock slope configuration is reached (Schoof 2007; Gomez et al. 2010). Once this instability, or tipping point, begins for a portion of the ice sheet, it further commits the Earth to future sea-level rise globally. Sea-level rise gradually inundates land, increases coastal erosion, elevates storm surges, and contributes to enhanced estuarine and riverine flooding (Glavovic et al. 2022). Changes in distribution or stratification of oceanic heat could rapidly fill the cavity beneath an ice shelf with relatively warm Circumpolar Deep Water instead of relatively cold water, thinning the ice shelf, reducing its ability to buttress upstream ice and thereby shift previously stable glaciers into unstable retreat. This effect is enhanced when warmer ocean water protrudes under partially-grounded ice (Bradley and Hewitt 2024).

Locally, increased melting of ice shelves adds freshwater, nutrients, and large amounts of particles to nearby surface waters (Meredith et al. 2018). This release changes the local primary production by changing light conditions and adding nutrients to the local waters. An additional effect of ice shelf collapse is iceberg scouring, or iceberg grounding, which occurs across the continental shelf, damaging sensitive benthic communities. New ice-free marine areas following ice shelf collapse become open to colonisation by benthic and pelagic organisms—effectively novel ecosystems (Brasier et al. 2021).

#### Ice sheet trends

Between 1992 and 2020, the Antarctic Ice Sheets (AIS) contributed 0.25 mm/yr to global-mean sea-level rise, about ~ 10% of the total rise over this period (approximately 92 ± 18 Gt/yr to sea level) (Otosaka et al. 2023). A significant acceleration has also occurred since the 1990s (Gardner et al. 2018; Rignot et al. 2019). This acceleration of Antarctic glacier melt is largely a result of increased warm water entering ice-shelf cavities and thinning ice shelves (Paolo et al. 2015).

A number of the major Antarctic glaciers possess unstable configurations, most notably in West Antarctica, which have been thinning and speeding up in recent decades under enhanced ocean forcing (Schmidtko et al. 2014; Smith et al. 2020). These changes have led to the suggestion that glaciers within West Antarctica’s Amundsen Sea Embayment may be in their early phase of unstable retreat (Joughin et al. 2014; Favier and Pattyn 2015; Pattyn and Morlighem 2020). Such a collapse would eventually produce a melt of ice equivalent to approximately 3 m of global-mean sea-level rise (Feldmann and Levermann 2015).

The much larger East Antarctic Ice Sheet (EAIS) also contains glaciers in unstable configurations in the Wilkes, Aurora, and Recovery basins (Fretwell et al. 2013). However, projections of the future of the ice sheet and the timing of the commencement of tipping points are very uncertain in this region due to limited knowledge of the physical state of the systems (Stokes et al. 2022).

### Ocean acidification

The Southern Ocean is the largest sink of anthropogenic carbon dioxide (CO_2_) due to low water temperatures, high primary productivity, and unique ocean circulation. This allows it to sequester large amounts of organic and inorganic carbon into the deep ocean. However, this also makes it particularly sensitive to ocean acidification. Ocean acidification in this region threatens one of the most iconic ecosystems globally, as well as its ability to sequester carbon, a critical ecosystem service that has a far-reaching footprint on global climate change (Watson et al. 2014).

Models show that extreme climate change, slowing down ocean currents and increasing rainfall, could produce a top layer of warmer, fresh water that does not mix with the cooler deeper waters and prevents carbon absorption (Chikamoto et al. 2023). This feedback loop could suddenly reduce the oceans’ ability to absorb carbon and make it irreversible in human time scales.

Ocean acidification also reduces the number of carbonate ions available, making it more difficult for marine organisms to calcify shells and skeletons. This will decrease their capacity to adapt and change community composition, potentially impacting both ecosystem structure and function. Changes to the food web are a likely way that this chemical change will express itself as a tipping point. The Antarctic ecosystem is highly reliant on species sensitive to acidification, including large diatoms, pteropods, krill, and other invertebrates (Hancock et al. 2020). The loss of these basal species at the base of the food web would impact species at higher levels, from ecologically and economically important species like krill (Trebilco et al. 2020) to iconic top predators that are a focus of tourism. The $820 million per year Antarctic tourism industry and the $370 million fishing industry (Stoeckl et al. 2024) would face steep transition costs and an uncertain future. More broadly, the regional ecosystem would be severely challenged as would carbon sequestration, a critical ecosystem service that has a far-reaching impact on climate change (Watson et al. 2014).

#### Ocean acidification trends

Rates of calcification in marine organisms declined in the Southern Ocean by 3.9% ± 1.3 between 1998 and 2014 (IPCC 2021). Earth system models show that undersaturation events with respect to aragonite will spread rapidly in the future, affecting ∼30% of A&SO surface waters by 2060 and > 70% by 2100. Undersaturation events are also predicted to extend in duration from 1-month, initially, to 6 months per year by 2050.

### Ocean circulation

A&SO creates some of the densest waters in the world. This cold and salty water sinks to the deepest part of the ocean, beginning the global ocean circulation process (Li et al. 2023). This circulation process cycles heat, carbon, oxygen, and nutrients across all the major ocean basins, globally (Purkey et al. 2018).

The densest water is the Antarctic Bottom Water, which is important to the physical ocean environment, especially in waters deeper than 4 km (Purkey et al. 2018). However, it is the northward subducting waters (e.g. Antarctic Intermediate Water and Subantarctic Mode Water) that are crucial to the global ocean biosphere (Li et al. 2022). These currents deliver nutrients heat, and salt globally, supporting roughly 75% of biological production north of 30°S (Sarmiento et al. 2004). Should the export of nutrients from A&SO slow down (possible if the polar nutrient trapping seen in multiple earth systems models under high emissions scenarios occurs (Moore et al. 2018)), it would take decades, if not centuries, for the loss of nutrients to be seen around the world. This means that ecosystem production and related human activities, including fisheries and tourism, would experience major declines beginning with areas closest to the Antarctic and spreading northward (e.g. Australia would likely see a major drop within decades (Solodoch et al. 2022)). Models indicate that, even without a complete slowdown, there is a projected potential of a 25% drop in global primary production within 250 years, leading to a 20–60% drop in potential fisheries production, depending on the region (Moore et al. 2018).

#### Ocean circulation trends

A recent model-based study found that oceanic circulation could undergo a substantial slowdown with increased Antarctic meltwater (Li et al. 2023). As much as a 40% slowdown could be seen by 2050, a relatively short period of time given the size of the current systems involved. Observations are in line with model predictions, showing a reduced abyssal overturning and ventilation of the Australian Antarctic Basin since 1994 (Gunn et al. 2023).

### Species redistribution

Increasing temperatures across the global oceans are driving species redistribution (Pecl et al. 2017). This is extending the range of temperate species towards higher latitudes (Meredith et al. 2022). The Southern Ocean is already seeing reductions in the extent of suitable habitats for cold-adapted species and, long term, the potential extinction of cold-adapted marine species (e.g. sessile benthic species, cold-adapted fish, krill, and other ice-dependent species). For example, under business-as-usual greenhouse gas emissions, 80% of emperor penguin colonies are projected to be quasi-extinct by 2100 (Jenouvrier et al. 2020). The current rate of habitat change is exceeding the evolutionary capacity to adapt. This leaves relocation as the most effective coping mechanism. However, polar species have nowhere to go.

Loss of species restructures local communities and their function, as does the entry of new species from elsewhere. Species shifting into polar habitats in the Southern Ocean may alter food webs and displace existing species (e.g. potential for Patagonian toothfish to displace Antarctic toothfish (Meredith et al. 2022)). In terrestrial Antarctica, increases in ice-free areas lead to increased connectivity of habitats and can result in increased homogeneity of biological diversity (Lee et al. 2017). Specific taxa may be facing extirpation if not extinction, reshaping the composition of the Antarctic and Southern Ocean ecosystems. Entire new ecosystems, or novel ecosystems, delivering different ecosystem services may result from a new mix of polar, sub-polar, and temperate species and how they interact in the changed physical conditions.

Even if the novel ecosystems stabilise quickly, geopolitical implications are possible. For example, it opens new challenges for managing transboundary fish stocks, either because they move out of national waters to become transboundary or because their extent goes beyond their previous jurisdictional boundaries (Pinsky et al. 2018). This could stress the Antarctic Treaty System (ATS) through potential conflict with other regional fisheries management organisations. Species redistribution also has the potential to escalate tensions, through “fishing wars”. While the active involvement of armed Navy vessels is more common in national waters (e.g., off Iceland), the potential remains. Nevertheless, confused or delayed management arrangements could open the door further to Illegal, Unregulated and Unreported (IUU) fishing in the region, a persistent concern even with only a single management body (Nilsson et al. 2016).

#### Species redistribution trends

Southward range shifts in the Southern Ocean have so far only been detected for Antarctic krill (Atkinson et al. 2019). Range changes have been observed for some species of sea birds and marine mammals due to changes in sea ice habitats and food availability (Bestley et al. 2020). Future declines in sea ice extent and the associated changes in prey distribution (Henley et al. 2020) are likely to drive the foraging areas of sub-Antarctic sea birds and marine mammals further south, resulting in increased energetic costs of foraging during the breeding season (Bestley et al. 2020; Hindell et al. 2020).

### Invasive species

Rising temperatures also open Antarctica to invasive species, or species introduced directly due to local human activity. This may include shipping, tourism, and scientific exploration (McCarthy et al. 2019) or through long-distance biologically mediated travel, for example on non-native kelp rafts (Avila et al. 2020). Modelling suggests that (1) climatic conditions no longer pose a barrier to entry on sub-Antarctic islands (Duffy et al. 2017), (2) ecological isolation will be broken by storm-driven dispersal and warming (Fraser et al. 2018), and (3) continental climatic barriers will weaken as warming continues across the region (Lee et al. 2017). In the terrestrial environment, the expansion of ice-free areas will increase the total land area available for non-native species. Once introduced, invasive species may be difficult to remove. For example, the European grass Poa annua, an invasive and disturbance-tolerant plant, has been eradicated from multiple locations on the Antarctic Peninsula. However, eradication has been less successful on King George Island, although efforts are ongoing (Bergstrom 2022).

Rats on the island of South Georgia (Pye and Bonner 1980) have been difficult to exterminate from the island. Antarctica is much larger, posing a larger challenge.

Growing numbers of invasive species can drive rapid change in terrestrial and marine ecosystems in A&SO (Grant et al. 2021). Invasive species may compete with, or directly impact endemic species. Past a certain threshold, enough new species will precipitate a new ecosystem state (as discussed above for species redistribution).

#### Invasive species trends

In the marine environment, invasive benthic invertebrates and macroalgae have already been detected off the Antarctic Peninsula and sub-Antarctic islands (Fraser et al. 2018; Avila et al. 2020; Brasier et al. 2021). Projected environmental changes will favour the further spread of invasive marine species in the future (Fraser et al. 2018). This is also true in terrestrial systems, where non-native species are a major driver of biodiversity change (Frenot et al. 2005; Chown et al. 2012b; McClelland et al. 2018). Despite the ATS, and sub-Antarctic management rulings restricting the introduction of non-native species to Antarctica and sub-Antarctic islands (de Villiers et al. 2006), alien species continue to arrive (Hughes et al. 2015). Currently, 14 non-native terrestrial species are recognised as having colonised the Antarctic Treaty region (Hughes et al. 2015), with approximately 200 species recognised as invading the sub-Antarctic islands (Frenot et al. 2005).

### Permafrost

Antarctic permafrost thawing represents a low-risk, high-impact tipping point. Permafrost refers to soil or sediment that remains at or below freezing temperatures (0 °C) for two or more consecutive years (Van Everdingen 2005). Mostly located along the Antarctic coast and sub-Antarctic islands (Hrbáček et al. 2023), Antarctic soils have been frozen for millennia and as they thaw with climate warming, viable viruses, bacteria, fungi, and other microorganisms are released (Wu et al. 2022). These represent possible pathogens that could spread across polar food webs or enter human populations. These microbes have been locked away for thousands of years, meaning most species (including humans) have never been exposed to them (da Silva et al. 2019). This presents a potential for pandemic disease outbreaks and potential deaths, as seen in the Arctic, where thawed ancient anthrax spores led to a death in 2016 (El-Sayed and Kamel 2021; Yarzábal et al. 2021).

#### Permafrost trends

Recent studies show the presence of significant concentrations of CO_2_, methane, and helium (up to 3.44 vol%, 18,447 ppmv and 6.49 ppmv, respectively) released at the interface between the permafrost and active layer in the McMurdo Dry Valleys (Ruggiero et al. 2023). This work suggests that soils have already begun to degas in some Antarctic regions and may extend to all of the 24,000 km of coastline of the Antarctic continent in the future. This positive feedback effect could amplify regional contributions to climate change gases. The lack of long-term observations makes it difficult to predict a time frame for such a methane-based tipping point.

### Pollution

As one of the last relatively pristine environments in the world, Antarctica is often thought to be particularly sensitive to pollution. Due to the low temperatures in the environment, contaminants are subject to slower natural processes of degradation and can accumulate and persist in the system. For example, hydrocarbon contamination from fuel spills in the soil degrades in months in a temperate climate, but takes years in Antarctica (Snape et al. 2006). A large enough pollution event could do irreparable damage to the unique environment of the A&SO.

The main local sources of pollution are leftover waste from abandoned stations and waste management practices put in before the adoption of the ATS Environment Protocol in 1998. In addition, contemporary issues of accidental fuel spills, fuel combustion, waste incineration, and sewage disposal also contribute as sources of pollution in Antarctica as they do globally (Tin et al. 2009; Klein et al. 2014; Chu et al. 2019). In addition, contaminants enter the A&SO through atmospheric deposition and ocean currents (Morley et al. 2020). This results in a range of contaminants in Antarctica’s terrestrial, nearshore marine, and Southern Ocean environments including metals, hydrocarbons, Persistent Organic Pollutants, microplastics, and nutrients. Contaminants initially impact species lower in the food web, altering communities, as well as bioaccumulating and causing impacts to higher-order predators (Cunningham et al. 2020; Palmer et al. 2021). This impacts the entire ecosystem (Caruso et al. 2022). It also has potential social implications on fisheries, tourism, and the ATS as iconic and fished species may become scarcer.

Research stations discharge sewage to be diluted and dispersed in the receiving marine environment (Smith and Riddle 2009). Human sewage contains a range of contaminants (e.g. metals, nutrients, pharmaceuticals) and non-native microbiota (e.g. enteric bacteria, viruses, parasites). The introduction of non-native organisms, which can carry pathogens across species, causes significant risk to native biota (Smith and Riddle 2009). In addition, impacts on benthic communities, bioaccumulation, histopathological abnormalities, and antibiotic resistance mechanisms in invertebrates and fish have been found within close proximity to sewage outfalls (Stark et al. 2016).

Marine litter is increasing in the Southern Ocean as it is transported from other parts of the world and discarded overboard from ships, especially fishing vessels (Lacerda et al. 2019). Plastics have been recorded in Antarctica since the 1980s, and microplastics have become more widespread within A&SO biota and the overall environment (Caruso et al. 2022). Plastic debris is a significant ingestion risk to wildlife, leading to starvation, death and breeding failures, particularly in seabirds (Ibañez et al. 2020).

#### Pollution trends

The ATS Environmental Protocol provides guidance regarding current waste disposal and management practices. However, the restrictions on bringing polluting materials to Antarctica and the requirements for the clean-up of waste from past activities are only loosely defined. This means that impacts from pollution and contamination continue to occur. Trends in emerging contaminants in Antarctica are of particular concern, with some now reported to be at levels close to that of highly populated regions in Europe and the USA; e.g. the discharge of the flame retardant chemical HBCD at McMurdo station (Chen et al. 2015). HBCD has also now been found to be accumulating in penguins, fish, sponges, and marine worms living in the surrounding area, impacting brain development and metabolism. Certain contaminants, recently found to be in excess of international environmental guidelines (including metals, hydrocarbons, and PCBs in marine sediments adjacent to Casey station), pose long-term ecological risks to local marine ecosystems (Stark et al. 2023).

Associated with increased shipping activity and tourism activities is the risk of oil spills. In recent years, several tourist and fishing ships have grounded in Antarctica’s poorly chartered waters, releasing diesel, lube oil, and gasoline into the nearby waters (e.g. M/S Nordkapp in Whalers Bay, M/V Explorer in Bransfield Strait (Wright 2008)). These kinds of spills can be catastrophic due to the abundance of marine life and the remoteness of A&SO preventing timely access required for intervention and/or clean-up.

### Antarctic treaty system

The ATS, comprising the Antarctic Treaty and associated instruments, governs human activity in A&SO. It ensures sustainable exploitation of Antarctica’s resources and that the continent is utilised for peaceful and scientific purposes. The ATS also regulates sewage and waste storage and disposal, as well as manages the removal of certain types of hazardous and biological waste. This means that the risk of pollutants impacting the environment from activities on the continent is managed within the ATS. Further, this Environment Protocol prohibits the discharge of oils, plastics, noxious liquid substances, and other chemicals considered harmful to the marine environment. Shifts in global geopolitics and the exacerbation of environmental pressures discussed above put the ATS under greater pressure and weaken its efficacy. This, in turn, could add to the governance challenges in managing the living and non-living resources in the A&SO. Reduced management of A&SO could produce undesirable outcomes for Antarctica and the planet.

Without strong management of human activities in the A&SO, countries or multinational companies prospecting and extracting valuable resources could undermine the ban on minerals extraction, currently prohibited in Antarctica. However, geological studies suggest that deposits of valuable mineral resources are likely to exist in Antarctica (Crispini et al. 2011). With the combination of retreating sea ice, ice sheets, new drilling techniques, and escalating scarcity-driven mineral prices (Talalay and Zhang 2022), costs may no longer be an insulating factor. The discovery of particularly rare earth minerals could entice individual nations to put pressure on the ATS, especially if access to mineral resources is limited elsewhere in the world. This may lead to mineral extraction in the A&SO and major disruptions to this environment. If A&SO ecosystems were to lose some of their protection due to mineral extraction, vulnerable species would likely become depleted, threatening the integrity of the terrestrial and marine ecosystems in the region.

A worst-case scenario is that one or more nations ignore the agreements in place around A&SO. This could happen by initially ignoring ATS regulations around shipping, bioprospecting, tourism, fisheries, and other critical activities (Liggett et al. 2017) and extend to ignoring core ATS values through enhanced militarisation and assertions of sovereignty (Brady 2017; Hamilton 2018). While it would be difficult to quantify the probability of such actions, and avoiding this scenario is paramount, they are all threshold behaviours that could tempt other nations to follow suit rather than support the ATS.

The realisation of this latter scenario could accelerate, if not tip, some of the other elements discussed in this paper. For example, the current tourism oversight that falls on industry bodies or individual nations could weaken or disappear in certain instances. Bases, human settlements, and other activities would be entirely regulated under national environmental regulations, which vary considerably. These changes would jeopardise protections around minimising impact and accessing protected areas through increasing pollution, introducing invasive species, and overall degrading the unique terrestrial and marine environments.

### Trends in the Antarctic treaty system

The ATS has already navigated geopolitical tensions, including those stemming from illegal fishing, demands for greater international participation in governance, negotiation of a minerals regime, growing pressure from climate change, and increasing and diversifying tourist activities. The situation in Antarctica has become more complicated in recent decades as significantly more countries are operating within the region. While positive in many ways, this adds complexities to negotiations between the Antarctic Treaty Consultative Parties (ATCPs). This has led to an increased failure to reach consensus during the Antarctic Treaty Consultative Meetings (ATCMs). Such tensions weaken the ability to navigate uncertain futures and respond to upcoming challenges, including the tipping points discussed in this paper. This has already been seen with the ATCPs’ inability to advance and strengthen environmental protection measures, especially in light of increased human activity in the region (Bastmeijer et al. 2023). This might indicate a shift in coalitions and power dynamics (Monteleone 2015).

An increase in dual-purpose activities has been seen on national bases. This includes the installation of satellite receiving stations, Global Navigation Satellite System ground stations, and continued deployment of military personnel and technology to support station logistics (Hemmings 2017; McGee et al. 2022). These activities, although currently used for scientific research, could quickly be utilised for other, more militaristic purposes.

## Interacting tipping points

**Fig. 3:**
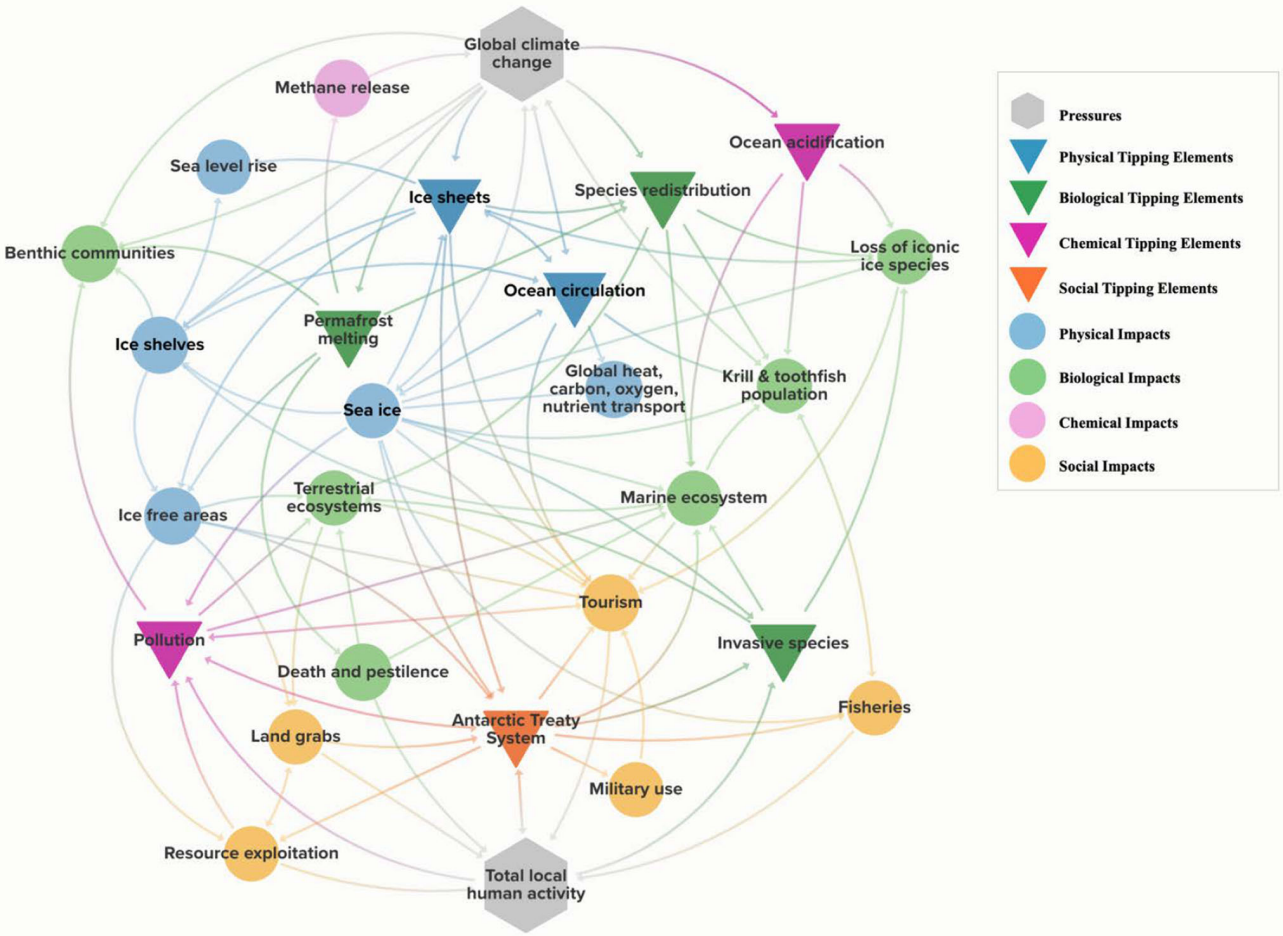
The interconnectedness of the Antarctic and Southern Ocean tipping points. This diagram shows the pressures (hexagons), tipping elements (triangles, orange for social, dark green for biological, dark blue for physical, purple for chemical), and impacts (circles). The connection arrows provide a directionality of influence. For example, the arrows specify which elements impact or are impacted by which other elements

The tipping points discussed in this paper are caused or augmented by human activities, either at the global or local scale (Fig. 3). The primary control variable for five of the tipping points is determined at the global scale, specifically the impact of climate change on ocean and air temperatures. The primary drivers of the other three tipping points focus instead on local human activities in the A&SO. However, these drivers do not exist in isolation. For example, scientists and tourists risk bringing invasive species onto the continent. However, many of these invasive species would not be able to survive in Antarctica without warming due to climate change.

The interaction between the global and local forces ensures that all the tipping elements in A&SO are experiencing multiple pressures. This means that a shift in any of the existing tipping elements or pressures will have impacts on all the tipping elements. Thus, if one of the tipping elements does tip, it may cause an acceleration, or cascade, of the other tipping points (Fig. 3).

**Fig. 4:**
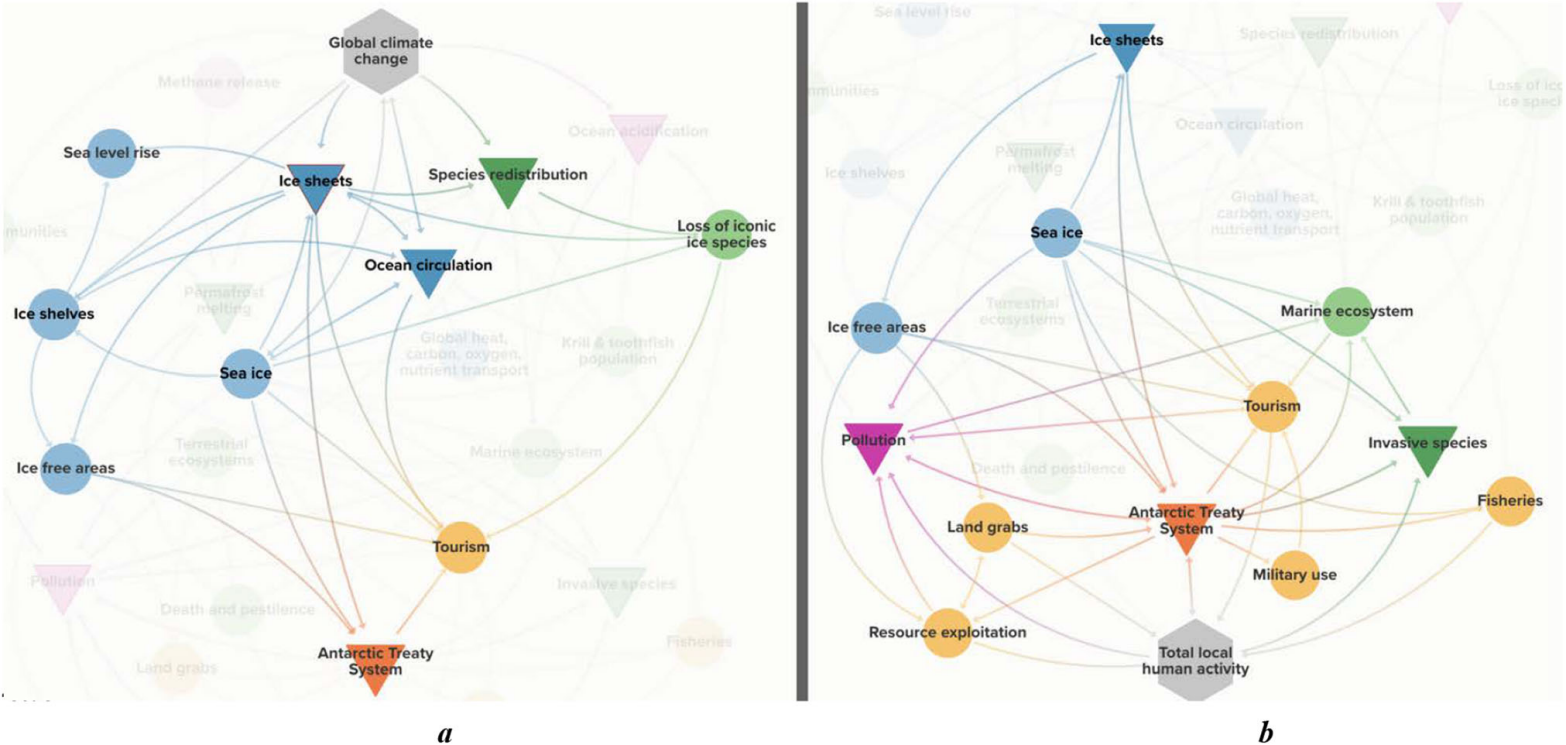
The connections of the ice sheets (**a**) and the Antarctic Treaty System (**b**) tipping points. This figure highlights the direct influences on and from two tipping elements, ice sheets and the Antarctic Treaty System. These are highlights pulled out of Fig. 3

For example, if any of the Antarctic ice sheets tip, this will put significant pressure on many of the other tipping points (Fig. 4a). Directly, it will alter global ocean circulation and enable additional species redistribution. It will also put significant strain on the ATS as new ice-free marine areas appear following ice shelf collapse. This will change fisheries, tourism, and access around the A&SO. Indirectly, the tipping of any of the Antarctica ice sheets will lower the albedo, leading to further melting in a positive feedback loop (Box et al. 2012). This will all contribute to climate change and put additional pressure on the other tipping points.

The interaction between the tipping elements is especially important to the functioning and resilience of the ATS. If the continental ice continues to melt around Antarctica, exposing more ice-free areas, the topography and accessibility to resources will change, leading to a complex interplay of factors affecting human activity on the continent. While the decision to establish bases in Antarctica is influenced by various socio-economic and geopolitical considerations, an increase in bases could pose challenges to the continent's pristine environment and political stability. This could lead to increased pollution, increased accessibility to minerals, and the demise of iconic species, all of which could result in the detriment of Antarctica’s unique wilderness character. These developments would put significant pressure on the ATS to continue managing access to Antarctica for only peaceful and scientific purposes.

This can in turn put additional pressure on the other tipping elements in the A&SO (Fig. 4b). The ATS has little, or no, direct influence on global climate change as it applies only to activities below 60° South. However, its continued functioning and resilience are critical in ensuring that the impacts of climate change in the region are minimised to the extent possible. Strengthening the existing ATS environmental protection and conservation measures will also reduce the likelihood of sudden direct and indirect shifts in tipping points (Stoeckl et al., In Press), For this to occur, the ATS must better recognise the interconnections between the A&SO and the rest of the world in order to protect Antarctica.

The importance of each individual tipping point is well understood (Schoof 2007; Tin et al. 2009; Watson et al. 2014; Klein et al. 2014; Duffy et al. 2017; Liggett et al. 2017; Purkey et al. 2018; Chu et al. 2019; Meredith et al. 2022; Wu et al. 2022; Li et al. 2023). However, the interactions and the cascade effects could magnify the impacts significantly and lead to multiple tipping points occurring (Fig. 4a, b). In addition, the A&SO tipping points may lead to the creation of new tipping points. For example, at present, fish populations, and hence fisheries, are not yet approaching tipping points. However, if climate change tips some of the current tipping elements, like species redistribution or ocean circulation, fish populations are likely to start approaching tipping points as well. This is especially true for krill, a critical part of not only the Antarctic marine ecosystem but also the global marine ecosystem (Cavan et al. 2019). Krill are a major food source for migrating fish and marine mammals, like whales which, in turn, affect marine ecosystems from Antarctica to the equator. Krill also plays an important role in mitigating climate change. By feeding on carbon dioxide-absorbing phytoplankton, krill store that carbon in their exoskeletons. Once shed, that exoskeleton, and hence the carbon, drops to the sea floor where the carbon is stored away (Cavan et al. 2019). On the social side, toothfish and krill fisheries make up the majority of all Antarctic fisheries, worth approximately $370 million per year (Stoeckl et al. 2024).

Although sea ice is not a tipping point in itself (Notz 2009; Wagner and Eisenman 2015), it puts pressure on many of the tipping points. For example, if the extent of the sea ice diminishes below a certain level, ice shelves will become vulnerable to wave action, potentially leading to their instability. Changes in sea ice will also lower the albedo and increase global warming, slow global ocean circulation, further enable species redistribution, and increase access to tourism and fisheries. Changes in sea ice would also affect the length of the fishing season and its economic viability (Nicol et al. 2012). Rising human activity may bring additional invasive species and pollution, adding further pressure on the Antarctic Treaty System (ATS) (Fig. 4a). If sea ice diminishes, it could directly lead to the acceleration towards, or the tipping of, six tipping elements. Indirectly, increases in global warming due to sea-ice loss, could also increase the risk of tipping two other elements: permafrost and ocean acidification. Hence, with sea-ice loss, all eight tipping points are potentially accelerated, either directly or indirectly.

It is difficult to predict all the indirect consequences of the Antarctica tipping points on the global physical, biological, chemical, and societal systems. However, we know that the impacts will be significant and, based on what we are seeing happening in the Arctic, different sectors of society across the globe will be differentially affected (Lenton 2012). Current estimates of the value that A&SO provide to the world through its regulation of climate and oceans is around $160 billion annually, almost surely an underestimate (Stoeckl et al. 2024). Critically, the ‘poor’ of humanity are often those who live in areas that are most likely to be impacted and are most often those with the least capacity to adapt. The tipping points are thus likely to exacerbate existing income inequalities—an added danger being that these inequalities become large enough to manifest as a new tipping point.

Because A&SO is a part of the biological and physical system, changes within the local environment can have direct and indirect impacts on the global environment and society. Therefore, it is important to consider the direct impacts, the interconnections among the tipping elements, and the human responses, adaptations, and consequent impacts of those adaptations.

## Conclusion

Antarctic tipping points represent significant dangers to global society. The possibilities for these tipping points to interact and cascade represents a potentially large but understudied, and underappreciated danger. We described several of the major Antarctic tipping points, including the physical, biological, chemical, and social, and highlighted how they are interconnected. We have also shown how critical the ATS is to manage and minimise the impact of the tipping points. A weakened, or ignored, ATS could significantly accelerate the other tipping points. As with climate change impacts, erosion of adherence to the ATS, if it were to occur, would result in negative impacts on Antarctica and the planet.

These conclusions only emphasise the need for more urgent action on climate change and continued resilient stewardship of the environment in the A&SO. To do that will require changing our economic paradigm, which is pushing us towards irreversible tipping points, to one that prioritises sustainable wellbeing of humans and the rest of nature. Only then will be able to sufficiently reduce human impacts, stay within planetary boundaries, and avoid cascading Antarctic tipping points.
